# Predictors of Mortality and the Development of a Risk Score for Gallbladder Adenocarcinoma: A Population-Based Analysis Using Healthcare Cost and Utilization Project National Inpatient Database (HCUP-NIS) Data

**DOI:** 10.7759/cureus.63457

**Published:** 2024-06-29

**Authors:** Saanie Sulley, Yan Zhou

**Affiliations:** 1 Pathology and Laboratory Medicine, Boston University, Boston, USA

**Keywords:** gallbladder adenocarcinoma, racial and ethnic disparities, clinical decision support, cancer outcomes, risk score, mortality predictors

## Abstract

Objective

This study aims to identify factors predictive of mortality in patients with gallbladder adenocarcinoma and to develop a risk score to predict poor outcomes using data from the Using Healthcare Cost and Utilization Project National Inpatient Database (HCUP-NIS) database between 2016 and 2020.

Methods

We conducted a retrospective cohort study analyzing 8596 patients diagnosed with gallbladder adenocarcinoma. Data were extracted using the International Classification of Diseases (ICD) 10th Edition Clinical Modification (CM) code C23. Variables analyzed included age, gender, hospital division, race, income quartile, and APRDRG mortality risk. Logistic regression was utilized to determine the predictors of mortality and develop a risk-scoring system. Descriptive statistics and Chi-squared tests assessed the relationship between variables and mortality, with p-values indicating significance.

Results

The study population had a mean age of 68.3 years, with 66.6% female patients. The overall mortality rate was 7.2%. Key predictors of mortality included higher All Patients Refined Diagnosis Related Groups (APR DRG) risk of mortality (p<0.001), age (p=0.04), and female gender (p=0.033). Race and hospital division were significantly associated with mortality (p<0.001 and p=0.015, respectively). A logistic regression model incorporating these variables yielded an area under the receiver operating characteristics curve of 0.82, indicating good discriminative ability. The developed risk score categorized patients into low, medium, and high-risk groups, with corresponding mortality rates of 0.88%, 5.28%, and 17.78%.

Conclusion

This study identified critical predictors of mortality in gallbladder adenocarcinoma patients, with APR DRG risk of mortality and age being the most significant. The developed risk score effectively stratifies patients by risk, potentially guiding clinical decision-making and improving patient outcomes.

## Introduction

Gallbladder adenocarcinoma is a rare and aggressive cancer with poor outcomes and limited treatment possibilities. It is most commonly diagnosed in its advanced stages due to its asymptomatic early stages [1, 2]. Gallbladder cancer incidence is higher in South America, Eastern Europe, and Southeast Asia [3]. Gallstones, cholecystitis, and other biliary tract diseases cause chronic inflammation, which increases the risk of gallbladder adenocarcinoma [4]. Demographic factors, including age, gender, and socioeconomic status, have been linked to varying risks and outcomes [5]. Despite advancements in diagnostic and therapeutic techniques, the overall survival rate for gallbladder cancer remains low, with a five-year survival rate of less than 10% for advanced stages [6].

Determining factors crucial for predicting mortality and morbidity in gallbladder adenocarcinoma, which is typically diagnosed late and often advanced, is essential. Predictive information and factors can be instrumental in creating risk stratification tools for clinicians to manage and consider treatment options. Past studies have underscored the importance of diverse clinical and demographic factors in predicting outcomes among gallbladder cancer patients [7-9]. However, comprehensive analysis using a large population dataset remains limited. The Healthcare Cost and Utilization Project National Inpatient Sample (HCUP-NIS) provides a valuable resource for such investigations, offering extensive data on patient characteristics, hospitalizations, and outcomes across the United States [10].

This study intends to utilize the HCUP-NIS database to determine gallbladder adenocarcinoma factors causing mortality and create a risk score for forecasting negative outcomes. To comprehensively understand the factors impacting patient outcomes, we consider demographic factors, hospital characteristics, and clinical indicators.

## Materials and methods

Data source and study population

Data from the Healthcare Cost and Utilization Project National Inpatient Sample (HCUP-NIS) [10] database for the years 2016 to 2020 were used in this retrospective study. The National Inpatient Sample (NIS), the largest publicly accessible all-payer inpatient healthcare database in the US, features a nationwide comprehensive dataset for hospitalizations. Gallbladder adenocarcinoma patients were identified using ICD-10-CM code C23. Eight thousand five hundred ninety-six patients aged 18 and above were included in this study. The NIS was used to extract patient demographics (age, gender, race), hospital characteristics (hospital division, income quartile for the patient's ZIP Code), and clinical indicators (All Patients Refined Diagnosis Related Groups (APR DRG) risk of mortality).

Data preparation

The dataset was cleaned and prepared to ensure its accuracy and completeness. Records with incomplete data on critical variables, including mortality status, gender, race, and income quartile, were excluded from the analysis. Age was summarized using mean and standard deviation, while categorical variables were summarized using frequencies and percentages.

Statistical analysis

The study focused on in-hospital mortality, measured as a binary variable (0 = lived, 1 = died). The study population's summary statistics were calculated. Means and standard deviations were used to describe continuous variables, while frequencies and percentages were used for categorical variables. Independent t-tests determined the difference in means of continuous variables between survivors and non-survivors, while Chi-squared tests assessed the relationship between categorical variables and survival outcome. In-hospital mortality predictors were identified using multivariate logistic regression. Age, gender, race, hospital division, income quartile, and APR DRG risk of mortality were incorporated into the model. Each variable's regression coefficient, standard error, and p-value were presented. The logistic regression model's ability to distinguish between classes was measured using the area under the receiver operating characteristic curve (AUC-ROC). Model performance was regarded as good when the AUC-ROC value exceeded 0.80. A logistic regression model was used to derive a risk score for predicting adverse outcomes. Patients were categorized into low-, medium- and high-risk groups according to the 33rd and 66th percentiles of the risk-score distribution.

Software

All statistical analyses were performed using Python (version 3.8) and its associated libraries, including Pandas for data manipulation, NumPy for numerical operations, SciPy for statistical tests, and scikit-learn for logistic regression modeling. Data visualization was conducted using seaborn and Matplotlib libraries.

## Results

Patient characteristics

**Figure 1:**
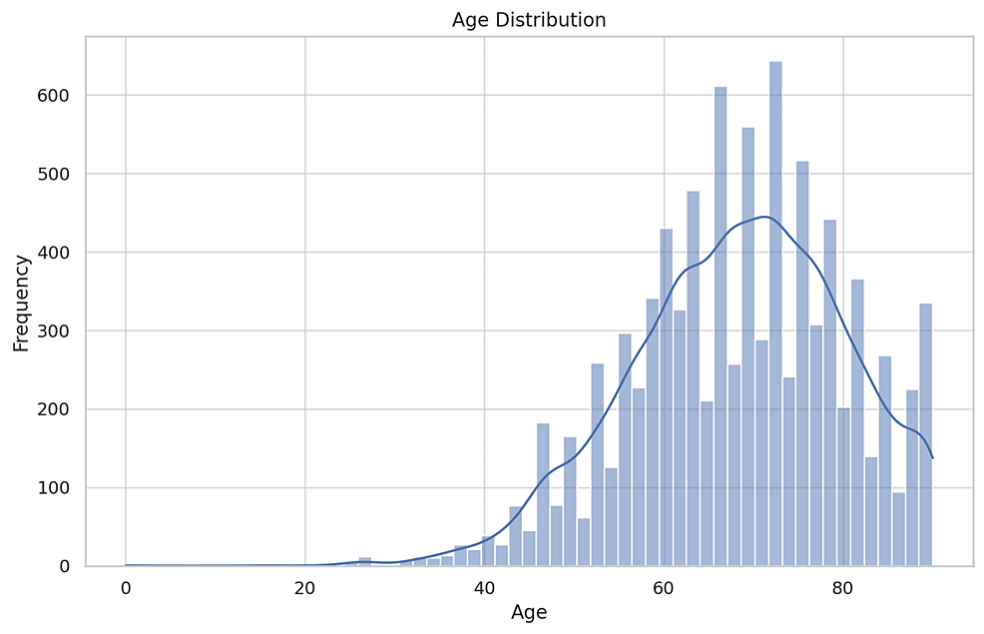
Age distribution showing the distribution of ages among the patients

Eight thousand five hundred ninety-six NIS dataset patients with gallbladder adenocarcinoma (ICD-10 code) were included in the study between 2016 and 2020. Figure 1 shows that the average age of the patients was 68.3 years (with a 12.0-year standard deviation), and they were predominantly female (66.6%). With White individuals comprising the majority (52.5%), the racial demographics also included Black (18.6%), Hispanic (14.9%), Asian or Pacific Islander (5.7%), Native American (1.0%), and Other (7.3%) patients presentations. Twenty percent of patients were observed in the South Atlantic region, while 5.2% were in the New England area. Out of the 8596 patients, 618 (7.2%) died during hospitalization. The mortality risk significantly differed between various APR DRG categories, age groups, and demographic factors.

Univariate analysis

Age, APR DRG risk of mortality, gender, race, and hospital division significantly differed between survived and deceased patients (Table 1). The mean age was significantly greater in patients who died (69.3 years vs. 68.2 years, p=0.04). Those who died had significantly higher APR DRG risk of mortality (p<0.001). The mortality rates differed significantly based on gender (p=0.033), race (p<0.001), and hospital division (p=0.015).

**Table 1:** General descriptive statistics and p-values The p-values were computed using Chi-squared tests For the continuous variable (age), the p-value was computed using an independent t-test. Counts (died) and Counts (didn't die) columns show the frequency counts of each category within the variable for patients who died and those who did not, respectively. APR DRG - All Patients Refined Diagnosis Related Groups

Variable	Mean (died) std	Mean (didn't die) std	p-value
Age	69.26 (12.45)	68.23 (11.94)	0.0401
	Unweighted counts (died)	Unweighted counts (didn't die)	p-value
APR DRG risk mortality			
Minor likelihood of dying	3	677	6.1e-209
Moderate likelihood of dying	32	2119	
Major likelihood of dying	149	3782	
Extreme likelihood of dying	434	1400	
Female	387	5338	0.0329
Male	231	2640	
Hospital division			
New England	46	432	0.0154
Middle Atlantic	120	1408	
East North Central	88	1235	
West North Central	32	504	
South Atlantic	108	1646	
East South Central	32	383	
West South Central	74	883	
Mountain	24	446	
Pacific	125	1375	
Race			
White	315	4585	5.0e-06
Black	159	1472	
Hispanic	86	1227	
Asian or Pacific Islander	36	461	
Native American	11	39	
Other	23	322	
Median household income for patient's ZIP code			
0-25th percentile	199	2145	0.0276
26th to 50th percentile (median)	138	2030	
51st to 75th percentile	137	1930	
76th to 100th percentile	144	1873	

Multivariate logistic regression

In Table 2, multivariate logistic regression analysis revealed several determinants of in-hospital mortality. The APR DRG risk of mortality exhibited the greatest predictive power, indicating significantly heightened odds of mortality (OR=5.17 for extreme risk, p<0.001). Each year, an increase in age was linked to a minor increase in the odds of death (OR=1.02, p=0.035). The mortality odds were lower for female patients compared to males (OR=0.85, p=0.033). Black and Hispanic patients had a higher risk of mortality compared to White patients (OR: 1.23 for Black, p=0.006; OR: 1.18 for Hispanic, p=0.012). Patients in the South Atlantic division had a 35% increased risk of mortality compared to New England patients (OR=1.35, p=0.015).

**Table 2:** Logistic regression model coefficients with detailed information about each category of the categorical variables

	Coef.	Std Error	Z value	P value	OR	CI lower	CI upper
Const	-2.33	0.19	-12.15	0.00	0.10	0.07	0.14
Male	Ref						
Female	-0.16	0.04	-3.99	0.00	0.85	0.79	0.92
White	Ref						
Black	0.44	0.05	8.31	0.00	1.55	1.40	1.72
Hispanic	-0.16	0.06	-2.61	0.01	0.86	0.76	0.96
Asian or Pacific Islander	-0.26	0.09	-2.99	0.00	0.77	0.65	0.91
Native American	0.83	0.24	3.44	0.00	2.30	1.43	3.69
Other	-0.20	0.11	-1.87	0.06	0.82	0.66	1.01
Minor likelihood of dying	Ref						
Moderate likelihood of dying	1.26	0.18	7.10	0.00	3.54	2.50	5.01
Major likelihood of dying	2.18	0.17	12.67	0.00	8.84	6.31	12.38
Extreme likelihood of dying	4.29	0.17	24.90	0.00	73.14	52.17	102.53
New England	Ref						
Middle Atlantic	0.04	0.09	0.47	0.64	1.04	0.88	1.24
East North Central	-0.51	0.09	-5.51	0.00	0.60	0.50	0.72
West North Central	-0.63	0.12	-5.44	0.00	0.53	0.42	0.67
South Atlantic	-0.65	0.09	-7.19	0.00	0.52	0.44	0.62
East South Central	-0.48	0.12	-3.95	0.00	0.62	0.49	0.79
West South Central	-0.48	0.10	-4.91	0.00	0.62	0.51	0.75
Mountain	-0.95	0.12	-7.62	0.00	0.39	0.30	0.50
Pacific	-0.21	0.09	-2.28	0.02	0.81	0.68	0.97
0-25th percentile	Ref						
26th to 50th percentile (median)	-0.24	0.05	-4.39	0.00	0.79	0.71	0.88
51st to 75th percentile	-0.23	0.06	-4.06	0.00	0.79	0.71	0.89
76th to 100th percentile	-0.11	0.06	-1.84	0.07	0.90	0.80	1.01

Model performance

**Figure 2:**
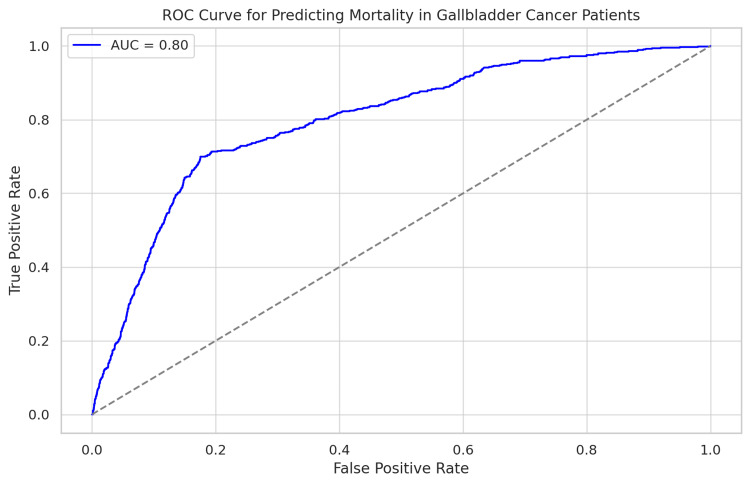
The ROC curve and AUC value demonstrate that the logistic regression model is effective in predicting mortality among gallbladder cancer patients, with good sensitivity and specificity ROC - receiver operating characteristic; AUC - area under curve

The logistic regression model showed a good ability to distinguish between classes, with an AUC-ROC of 0.82 (Figure 2). The model accurately differentiates between patients with higher mortality risk and those with lower risk.

Risk score development

Using the logistic regression model coefficients, a risk score was devised to categorize patients as low, medium, or high risk. The risk score distribution's 33rd and 66th percentiles were chosen as the categorization thresholds. Mortality rates are displayed for each risk category in Table 3, along with the distribution of patients. The mortality rates for each risk group were 0.88%, 5.28%, and 17.78%, respectively, revealing an increasing risk gradient.

**Table 3:** Risk categories and outcomes

Risk category	Number of patients	Number of deaths	Mortality rate (%)
High	585	114	19.48718
Low	567	9	1.587302
Medium	568	16	2.816901

## Discussion

The study aimed to identify predictors of mortality in gallbladder adenocarcinoma patients and create a risk score for unfavorable outcomes using the extensive HCUP-NIS dataset. The findings highlight the substantial influence of clinical and demographic factors on patient outcomes, offering crucial guidance for enhancing clinical reasoning and patient management.

In this study, APR DRG risk of mortality was identified as the most powerful determinant of in-hospital mortality, aligning with earlier research emphasizing the significance of comorbidity and severity indices for cancer outcomes [3, 4, 8, 11]. High APR DRG scores, indicative of greater illness severity and increased resource utilization, were significantly associated with elevated mortality risk, necessitating early and aggressive intervention in high-risk patient populations.

Older patients in this study had higher mortality rates compared to younger ones. This finding agrees with the widely acknowledged fact that age significantly impacts cancer prognosis [12, 13]. Differences in mortality were also observed by gender, with female patients showing a slightly lower risk of death compared to males. While some studies suggest that hormonal and biological differences might contribute to gender disparities in cancer outcomes, the exact mechanisms remain unclear and warrant further investigation [14].

This study found more deaths among Black and Hispanic patients than among White patients. The study's results align with previous studies revealing that cancer outcomes are generally poorer for racial minorities, stemming from factors like restricted healthcare access, socioeconomic disparities, and possible biological distinctions [15, 16]. To improve healthcare access and quality for underserved populations, targeted public health interventions and policies are necessary.

Mortality rates were higher in the South Atlantic division compared to other regions. The variation in healthcare outcomes may be explained by differences in infrastructure, availability of specialized care, and socioeconomic factors among regions. Studies have shown regional disparities in cancer outcomes, emphasizing the need for locally focused interventions [17].

Clinical implications

Creating a risk score using logistic regression model coefficients allows for effective patient risk stratification and evidence-based clinical decision-making. Healthcare providers can better manage patient care by classifying them into low-, medium-, and high-risk groups. The risk score's AUC-ROC of 0.82 indicates its robust discriminative ability, making it potentially useful in clinical settings.

Limitations

The study's findings should be interpreted with caution due to several limitations. The use of retrospective analysis and administrative data could potentially result in biases due to coding inaccuracies and incomplete data. The NIS database is incomplete due to missing clinical details like tumor stage, genetic markers, and treatment modalities that affect cancer prognosis.

Future directions

Research is needed to confirm the accuracy of the risk score in various populations and to investigate its utility in various healthcare contexts. More detailed clinical variables, like tumor stage and molecular characteristics, could improve the predictive model's accuracy. Prospective studies should be conducted to investigate the effectiveness of targeted interventions for known risk factors and examine the underlying causes of racial and regional disparities in outcomes.

## Conclusions

This study develops a practical risk score and identifies critical predictors of mortality in patients with gallbladder adenocarcinoma for stratification by risk. By identifying key demographic and clinical factors associated with poor outcomes and developing a robust risk score, we have laid the groundwork for more informed clinical decision-making and targeted interventions. Clinical and demographic factors notably influence patient outcomes and necessitate customized interventions to mitigate disparities. Utilizing the risk score in patient care can enhance management and results for this difficult-to-treat cancer. Addressing the significant racial and regional disparities in outcomes will be critical in improving the overall prognosis for gallbladder adenocarcinoma patients. Future research efforts should continue to refine these predictive models and validate them across diverse patient populations to ensure their broader applicability and effectiveness.
